# Hyperlipidemia Intervention from Medicine-Food Homologous Plants: A Review of Regulatory Status, Processing Techniques, and Lipid-Lowering Mechanisms

**DOI:** 10.3390/biology15141142

**Published:** 2026-07-13

**Authors:** Jian Chen, Daomin Liu, Peifeng Zhu, Lu Liu

**Affiliations:** Yunnan Yunzhong Institute of Nutrition and Health, College of Traditional Chinese Medicine, Yunnan University of Chinese Medicine, Kunming 650500, China; 13354630755@163.com (J.C.); kmliu@vip.163.com (D.L.)

**Keywords:** medicine-food homology, bioactive components, anti-hyperlipidemia

## Abstract

Background: The rising global prevalence of hyperlipidemia is closely linked to current dietary patterns, sparking interest in “medicine-food homology” (MFH) plants as natural resources that provide both nutritional benefits and therapeutic effects. This study reviews MFH plants used to treat hyperlipidemia, incorporating changes in regulatory mechanisms, the impact of processing on bioavailability, and evidence of their lipid-lowering effects. Methods: This narrative review, supported by a structured literature search, summarizes the practical applications of MFH plant species with both medicinal and edible properties and discusses their bioactive components and lipid-regulating mechanisms. Results: MFH plants not only contain anti-lipid active ingredients but also have improved bioavailability after processing through processing technology and function through lipid metabolism pathways and the gut-liver axis. Conclusions: The development and application of MFH plants face several challenges, including incomplete and inconsistent international regulatory frameworks, difficulties in quality control, and limited clinical evidence for lipid-lowering mechanisms. Nevertheless, MFH plants hold promise for development into functional foods or nutraceuticals aimed at supporting lipid regulation.

## Simple Summary

High levels of fats such as cholesterol and triglycerides in the blood are becoming increasingly common worldwide and can raise the risk of heart and blood vessel diseases. Plants traditionally used as both foods and medicines have therefore attracted interest as natural resources for dietary support and product development. This review summarizes how these plants are regulated in different regions, how processing methods may improve the stability and absorption of their beneficial ingredients, and what evidence supports their potential effects on blood fat control. The reviewed studies suggest that some plant ingredients may help regulate fat handling in the body, especially through actions involving the liver and the gut. However, the evidence is not equally strong for all plants or products. Differences in regulations, plant sources, processing methods, product quality, and the limited number of human studies remain major challenges. Overall, these plants may be useful for developing safer and better standardized functional foods, but stronger clinical evidence and quality control are needed before broad use in blood fat management can be recommended.

## 1. Introduction

Contemporary high-fat and high-sugar diets are emerging as potential triggers for numerous metabolic diseases, including hyperlipidemia, a major risk factor for cardiovascular diseases [1,2]. In response, there is increasing demand for natural, functional, and safe food alternatives [3,4].

Medicine-food homology (MFH) plants, possessing nutritional and medicinal properties, have been used in traditional diets and healthcare [5,6]. Bioactive components such as polysaccharides, flavonoids, and terpenoids possess diverse health-promoting activities, emerging as a primary study focus [7,8]. While the potential for MFH plants as functional in certain aspects is well recognized, many challenges remain. Variable regulatory criteria currently make it difficult to conduct a full review of their safety and efficacy. Furthermore, the effect of processing on the stability and bioavailability of bioactive components is understudied. Lastly, both the underlying mechanisms of their lipid-lowering effects and the supporting clinical evidence require further refinement [9,10].

Hyperlipidemia remains a major public health burden worldwide because it is closely associated with the development of atherosclerosis, coronary heart disease, stroke, and other cardiovascular complications. Although pharmacological lipid-lowering therapies are effective in many patients, long-term drug use may be limited by adverse effects, poor adherence, and concerns regarding chronic medication exposure [11]. In this context, MFH plants have attracted increasing attention as promising dietary resources that combine nutritional value with potential therapeutic benefit. However, the current evidence base is still fragmented, as most studies focus on isolated compounds, preclinical models, or single processing approaches, making it difficult to compare efficacy across plant species and to translate these findings into standardized applications.

By integrating regulatory differences, processing-related effects on bioavailability, and evidence on lipid-lowering mechanisms, this review aims to provide a coherent framework for understanding the role of MFH plants in hyperlipidemia-related functional food and nutraceutical development, thereby supporting their standardization and translational application.

## 2. Methodology and Literature Search Strategy

Although this is primarily a narrative review, a structured literature search strategy was used to enhance transparency, reproducibility, and methodological rigor. A PRISMA-informed workflow was used as a methodological reference for literature identification, duplicate removal, title/abstract screening, full-text eligibility assessment, and manual reference checking. Importantly, this review was neither designed nor registered as a formal systematic review, and no meta-analysis, formal risk-of-bias assessment, or GRADE evaluation was performed. Therefore, PRISMA served as a guide for reporting transparency, rather than to claim full systematic-review compliance.

The databases, including PubMed, Web of Science Core Collection, and ScienceDirect, were searched for studies published between August 2011 and April 2026. Google Scholar was additionally consulted as a supplementary source to identify additional relevant studies and citation-linked articles. The search strategy combined controlled vocabulary where applicable, such as MeSH terms in PubMed, with free-text keywords related to medicine-food homologous (MFH) plants, lipid metabolism, and processing technologies. Representative keywords included: “medicine-food homology,” “functional foods,” “hyperlipidemia,” “dyslipidemia,” “lipid metabolism,” “gut microbiota,” “gut–liver axis,” “AMPK,” “PPAR,” “SREBP,” “microencapsulation,” and “nanoparticles.” Boolean operators (AND/OR) were used to combine search terms. Additionally, reference lists of selected articles and key reviews were manually screened.

Studies were included if they met the following criteria: (1) they focused on MFH plants listed in the “Substances that Are Both Food and Traditional Chinese Medicinal Herbs” issued by the National Health Commission of the People’s Republic of China; (2) they provided original experimental or clinical evidence related to lipid-lowering effects, processing technologies, mechanisms of action, or authoritative regulatory/safety information relevant to MFH plant products; and (3) they were published in English or Chinese. Primary evidence included original in vitro studies, animal studies, and human clinical studies. Systematic reviews and meta-analyses were used mainly to contextualize the evidence base, identify additional primary studies, and support interpretation where appropriate.

Narrative and other non-systematic reviews were not treated as primary evidence, although they were consulted for background context, terminology clarification, and reference tracking. Studies were excluded if they lacked adequate controls, had insufficient description of the intervention, plant source, study model, or lipid-related endpoints, or reported outcomes not directly relevant to hyperlipidemia, lipid metabolism, processing technologies, or MFH product safety.

After duplicate removal, title/abstract screening, full-text eligibility assessment, and manual reference checking, 170 studies were included in the final narrative synthesis. The included literature was organized into three thematic domains: (i) regulatory status and safety considerations of MFH botanicals; (ii) processing technologies, including microencapsulation, colloidal carriers, and nanoparticles, aimed at improving bioavailability; and (iii) in vitro, in vivo, and human clinical evidence related to lipid-lowering mechanisms of MFH bioactive constituents. Where available, the extracted information included plant species, plant part, bioactive compound or extract type, processing or formulation method, study model, dose and duration, lipid-related outcomes, proposed molecular mechanisms, safety findings, and evidence category.

Given the narrative nature of this review, findings were interpreted critically with consideration of study design, experimental model, sample size, dose relevance, reproducibility of lipid-related outcomes, consistency across studies, and availability of human validation. In this review, “evidence category” refers to the primary study model and the availability of human validation rather than to a formal GRADE-based rating. Evidence from in vitro studies, animal experiments, and human clinical studies was distinguished throughout the manuscript to avoid overinterpretation of preclinical findings. Mechanistic findings derived solely from cell or animal models were interpreted as hypothesis-generating unless supported by human lipid endpoints.

## 3. Current Regulatory Status and Challenges for MFH Plants Under Different Regulatory Systems

Botanical ingredients are the main components of MFH substances and appear across foods, dietary supplements, and health products, but their regulatory classification and pre-market requirements vary substantially between jurisdictions [12]. This heterogeneity affects safety testing, labeling, contaminant control, batch testing, clinical-evidence requirements, and cross-border market access. A key limitation is that the same MFH plant may be regulated as a traditional food, dietary supplement, functional ingredient, novel food, or herbal medicinal product depending on jurisdiction and intended use. Therefore, regulatory classification should not be viewed merely as an administrative category but as a determinant of product-level safety assessment, manufacturing control, standardization, quality assurance, and post-market surveillance. For MFH products, regulatory status should therefore be interpreted together with product-specific safety, quality-control, and evidence requirements rather than as a simple indicator of efficacy or safety.

In China, the National Health Commission (NHC), together with the State Administration for Market Regulation (SAMR), has established the Catalogue of Substances Traditionally Used as Both Food and Chinese Medicinal Materials, which comprises 106 substances, including 93 botanical substances and 13 animal-derived substances [13]. Accordingly, the botanical entries, which account for 93 of the 106 listed substances, are the primary focus of this review. This catalog was developed based on Chinese traditional consumption patterns, dietary needs, international regulatory practice, pilot production data, and risk-monitoring evidence. Thus, traditional dietary use should not be extrapolated automatically to modern MFH products such as purified extracts, high-dose capsules, fermented preparations, or nanoformulations. These products require botanical authentication, plant-part verification, chemical fingerprinting, marker-compound quantification, contaminant testing, stability assessment, and batch-to-batch consistency evaluation.

In the United States, the Dietary Supplement Health and Education Act of 1994 (DSHEA) created a supplement-specific framework that distinguishes botanical ingredients from drugs [14]. Under DSHEA, dietary ingredients that were marketed in the United States before October 15, 1994, are generally not considered New Dietary Ingredients and may be used in dietary supplements without the Food and Drug Administration (FDA) pre-market approval, provided that the products are not intended to diagnose, treat, cure, or prevent disease [15]. Botanical ingredients lacking such historical use are generally subject to the New Dietary Ingredient (NDI) notification process, which requires manufacturers to provide safety information before market entry and submit a pre-market notification to the FDA [16,17,18]. A critical limitation of this framework is that historical marketing status may allow products with substantial compositional variability or limited modern toxicological evaluation to reach consumers, especially when concentrated extracts or novel formulations differ from traditional forms. Therefore, manufacturer-controlled quality assurance, contaminant testing, compositional standardization, and adverse-event monitoring are particularly important for botanical supplements.

In the European Union, Directive 2002/46/EC on Food Supplements and related national regulations impose additional requirements on botanical ingredients, including safety evaluation and, where appropriate, authorization through pathways such as the Novel Food Regulation or the Traditional Herbal Medicinal Products Directive, depending on intended use [18]. Despite the inclusion of 93 botanical plants in China’s MFH catalog, international regulatory alignment remains incomplete. Some MFH substances are recognized in general food or supplement contexts in regions overseen by authorities such as the European Food Safety Authority (EFSA) or Australia’s Therapeutic Goods Administration (TGA), whereas fewer meet specific functional food or purity requirements such as Japan’s “Foods with Nutritional and Functional Claims” (FNFC) system or Codex-related standards. This multiplicity of regulatory pathways creates uncertainty because similar MFH products may require different safety, compositional, toxicological, or human-evidence dossiers across jurisdictions. For products making lipid-related functional claims, evaluation should consider preparation method, daily intake, active-marker content, intended population, duration of use, product stability, and the availability of human safety and efficacy data, rather than relying only on historical use.

A major barrier to global regulatory harmonization is the lack of aligned safety thresholds and toxicology expectations for contaminants [18,19]. Many MFH plants contain variable levels of toxic elements, such as arsenic, cadmium, mercury, and lead, which may reflect differences in origin, processing, packaging, and transportation [20,21]. These persistent contaminants may bioaccumulate and pose health risks, particularly when exposure is prolonged or occurs in combination with other compounds [21,22]. Therefore, safety evaluation should be product-specific rather than plant-name-specific. In addition to toxic-element testing, modern MFH products should be evaluated for pesticide residues, mycotoxins, microbial contamination, residual solvents when extraction is used, and potential adulterants [23].

Recent advances in analytical chemistry, exposure assessment, and product standardization are gradually reducing these regulatory barriers [24]. However, these advances need to be translated into practical quality-control requirements, including verified botanical identity, defined plant part and processing method, chemical fingerprinting, marker-compound quantification, contaminant limits, stability testing, batch-to-batch consistency evaluation, and post-market adverse-event monitoring. Such measures would help ensure that safety and efficacy claims are linked to reproducible product quality rather than broad traditional-use categories [23,25]. 

Table 1 presents a representative subset of MFH plant species selected from the NHC catalog based on regulatory coverage, safety concerns, processing relevance, and published hypolipidemic evidence. The complete species list is provided in Table A1.

This table presents a representative subset of MFH plant species selected from the National Health Commission (NHC) Catalogue of substances traditionally used as both food and Chinese Medicinal materials because they are representative entries with available information on regulatory coverage, safety concerns, processing relevance, and/or published hypolipidemic evidence. The complete species list is provided in Table A1. The labels in the “Primary Basis for Inclusion” column are not mutually exclusive.

## 4. Advancing MFH Plants for Hyperlipidemia Management via Integrated Strategies

Diet can influence lipid metabolism through phytochemical–microbiome–liver interactions [26]. As natural sources of foods and dietary supplements, MFH plants provide promising resources for hyperlipidemia management when combined with modern food processing and delivery technologies [27]. However, the evidence supporting these integrated strategies varies substantially across traditional dietary use, formulation studies, animal models, and human trials. Therefore, this section distinguishes technological or formulation-level improvements from evidence showing direct lipid-lowering effects. Mechanisms supported by human lipid outcomes are regarded as relatively established, whereas those based only on animal, cellular, or formulation-level data are considered plausible but still require validation. Improved stability, solubility, bioaccessibility, or delivery efficiency is therefore considered supportive evidence, but not proof of hypolipidemic efficacy unless accompanied by lipid-related biological outcomes. Importantly, translation of advanced processing and delivery technologies remains constrained by scale-up reproducibility, residual-solvent and thermal-degradation risks, food-grade excipient selection, sensory and food-matrix compatibility, cost, batch-level characterization, and regulatory uncertainty for nano- or exosome-like platforms. These barriers must be addressed before formulation advances can be translated into clinically meaningful MFH products.

### 4.1. Synergism of MFH Plants in Traditional Consumption

Direct intake of MFH plants may provide both essential nutrients and protection of active ingredients through the natural food matrix [28]. This food-matrix effect is particularly relevant in traditional dietary contexts, where bioactive compounds are consumed together with starches, proteins, lipids, dietary fiber, and other phytochemicals rather than as isolated ingredients. Whole grains provide a representative example. Barley (*Hordeum vulgare* L.) is one of the widely cultivated cereal crops worldwide [29]. It contains β-glucans that interact with starch and other food components to slow gastric emptying, increase satiety, and modulate postprandial metabolic responses [30,31]. Among whole-food mechanisms, β-glucan-related LDL-C reduction is relatively well supported when confirmed by human lipid outcomes; by contrast, broader matrix effects such as satiety, digestion kinetics, and postprandial modulation should be considered supportive rather than proven lipid-lowering mechanisms unless lipid endpoints are measured.

Traditional dietary practices also illustrate how MFH plants and related food plants are incorporated directly into meals rather than consumed as isolated supplements. In the Basque Country of Spain, a “health-food” paradigm includes the use of specific plants as part of daily meals or post-meal dietary practices [32]. For example, *Juglans regia* (walnut) dietary interventions have reported reductions in LDL-C and total cholesterol, supporting the relevance of whole-food interventions for lipid management [33]. *Prunus spinosa* (blackthorn), traditionally consumed in the liqueur “Patxaran,” has been used after heavy or lipid-rich meals to support digestion [34]. Similarly, *Chamaemelum nobile* (Chamomile) and *Thymus vulgaris* (Thyme) are used as seasoning herbs and digestive aids in meal contexts [35]. Here, walnut-related LDL-C and total cholesterol reductions represent relatively stronger clinical evidence. In contrast, the digestive or post-meal uses of blackthorn, chamomile, and thyme remain traditional or observational evidence and should not be presented as proven lipid-lowering mechanisms.

Nutrient-dense wild plants provide another example of whole-food-based synergy. Purslane (*Portulaca oleracea* L.) contains vitamins, minerals, antioxidants, and ω-3 fatty acids that may collectively support metabolic health [36,37,38,39]. Collectively, these examples suggest that traditional whole-food consumption may provide broader metabolic benefits than isolated constituents alone through interactions among dietary fiber, phytochemicals, fatty acids, minerals, and the surrounding food matrix [40]. Nevertheless, traditional dietary plausibility remains context-setting evidence rather than direct clinical proof. Translation into commercial MFH products requires extraction standardization, active-fraction quantification, dose matching, stability testing, and confirmation that processing does not eliminate matrix-based benefits.

### 4.2. Optimization of Extraction Solvents: Modulating Phytochemical Profiles to Improve Lipid-Lowering Activity

Extraction solvent polarity influences the phytochemical profile of MFH plants and may thereby affect biological activity [41,42]. Polar solvents and aqueous decoctions preferentially recover polysaccharides, saponins, and hydrophilic phenolics, which are often associated with gut microbiota remodeling, short-chain fatty acid production, bile acid homeostasis, and intestinal barrier support [43,44]. Intermediate-polarity systems such as aqueous ethanol and ethyl acetate are more effective for flavonoids and polymethoxy flavones [45]. In citrus-derived materials, these fractions are frequently associated with anti-lipase activity and broader metabolic benefits. At the same time, recent reviews also link citrus polyphenols to the regulation of gut homeostasis and improvements in metabolic syndrome [46]. Nonpolar solvents tend to enrich sterols and triterpenoids, which may modulate bile acid metabolism, transport, and hepatic lipid handling [47,48]. Evidence from recent MFH studies highlights these solvent-dependent differences. For Chenpi, solvent fractionation and citrus polyphenol studies indicate that ethanol/ethyl acetate fractions can enrich polymethyl flavones and other bioactive constituents relevant to lipid metabolism [49,50]. For *Pueraria lobata*, the extraction literature shows that water-based and green-solvent systems can efficiently recover puerarin/isoflavones with improved bioavailability and lipid-regulatory benefits [51,52]. However, solvent-driven enrichment mainly provides phytochemical and preclinical evidence; it identifies candidate active fractions but does not itself prove clinically meaningful lipid-lowering.

However, solvent-dependent enrichment of bioactive constituents creates several translational challenges. Food-grade or green solvents should be prioritized; residual-solvent limits must be validated when non-aqueous solvents are used; and solvent recovery, waste disposal, extraction kinetics, and batch-to-batch chemical consistency must be considered during scale-up. Future solvent-comparison studies should combine phytochemical fingerprinting, residual-solvent testing, matched-dose controls, and lipid-specific endpoints in animal or clinical models. Only when solvent-defined fractions are linked to reproducible lipid outcomes should their molecular mechanisms be considered functionally validated.

### 4.3. Advanced Delivery Systems: Overcoming Bioavailability Barriers

Several lipid-lowering constituents from MFH plants are limited by poor aqueous solubility, low oral absorption, chemical instability, and potential gastrointestinal intolerance [53,54,55]. Encapsulation, colloidal carriers, and nanoparticle-based delivery systems can partially overcome these limitations by improving protection, dispersibility, release behavior, and bioaccessibility [56]. However, improved formulation performance is an intermediate technical endpoint. In most delivery studies, the directly demonstrated mechanism is improved delivery rather than confirmed lipid-lowering. A therapeutic claim is justified only when advanced delivery produces superior lipid-related outcomes, such as reductions in serum TG, TC, LDL-C, hepatic lipid accumulation, or lipid-metabolic gene/protein expression in vivo or in humans.

For clarity, delivery-related outcomes can be classified into three levels: formulation endpoints, including encapsulation efficiency, particle size, release profile, and stability; bioavailability-related endpoints, including in vitro bioaccessibility, gastrointestinal stability, and pharmacokinetic exposure; and lipid-efficacy endpoints, including serum lipid profiles, hepatic lipid accumulation, bile-acid metabolism, or validated lipid-regulatory biomarkers. Only lipid-efficacy endpoints provide direct evidence of hypolipidemic activity; formulation and bioavailability endpoints should be described as supportive or enabling evidence. Translation of advanced delivery systems also requires attention to regulatory classification, food-grade excipient status, allergen labeling, reproducible manufacturing, physicochemical characterization, altered ADME or herb–drug interaction risks, sensory properties, shelf life, cost, and safety or PK/PD data before human testing. 

#### 4.3.1. Core Principles of Encapsulation: Materials, Delivery Efficacy, and Preservation

Encapsulation remains a central strategy to render MFH bioactives usable in foods and nutraceuticals, especially for poor water solubility, low gastrointestinal stability, or limited oral bioavailability [57,58,59]. Common food-grade carriers include proteins, polysaccharides, lipids, and composite biopolymer systems. Protein-based wall materials, such as soy protein isolate, whey protein, and plant protein isolates, provide emulsifying and film-forming properties. Polysaccharides, including maltodextrin, gum arabic, pectin, alginate, chitosan, and inulin, contribute to matrix formation, digestive stability, and controlled release. Lipid-based systems, including liposomes, solid lipid nanoparticles, and nanostructured lipid carriers, are particularly suitable for lipophilic compounds [60,61,62,63,64,65]. Thus, the established mechanism of encapsulation is carrier-mediated protection and controlled release; its lipid-lowering mechanism remains uncertain unless improved delivery is linked to lipid outcomes. 

Beyond composition, delivery performance depends on encapsulation efficiency, particle-size distribution, surface properties, matrix interactions, digestive stability, and release kinetics. These parameters should be reported together with pharmacokinetic or metabolic readouts when enhanced hypolipidemic activity is claimed [66,67,68]. Encapsulation also preserves labile bioactives from oxidation, light, heat, and moisture during processing and storage [69,70,71]. Nevertheless, preservation or improved bioaccessibility should not be equated with lipid-lowering efficacy unless lipid-specific outcomes are demonstrated in vivo or in human studies [72]. Therefore, encapsulation should be presented as a validated formulation strategy, but not as a proven hypolipidemic mechanism by itself.

#### 4.3.2. Microencapsulation

MFH plant-derived oils are often rich in unsaturated fatty acids, making them susceptible to oxidation and rancidity [73]. Microencapsulation can improve oxidative stability, handling properties, and shelf life. Common wall materials include food-grade proteins, polysaccharides, lipid matrices, and composite systems; typical processes include spray-drying, freeze-drying, complex coacervation, and emulsion-based encapsulation [74]. Protein–polysaccharide blends are frequently used because they combine emulsification, film formation, oxidative protection, and controlled digestive release [75]. These mechanisms are established at the formulation level, but their contribution to lipid-lowering must be confirmed using metabolic endpoints.

Sea buckthorn (*Hippophae rhamnoides*) seed oil microcapsules were prepared using a soybean protein isolate–soybean polysaccharide blend (2:3) as the shell material under optimized conditions (160 °C, core-to-shell ratio of 1:3), improving encapsulation efficiency, antioxidant capacity, storage stability, and handling performance [76,77]. However, this example primarily demonstrates formulation-level improvement. It does not establish superior lipid-lowering efficacy unless the microencapsulated product is compared with free oil or non-encapsulated extract using serum lipid, hepatic lipid, or bile-acid endpoints in animal or human studies. 

#### 4.3.3. Colloidal Carriers

Colloidal carriers have been developed to improve the stability, dispersibility, and oral bioavailability of many MFH-derived bioactives [78,79]. Protein–pectin systems exemplify food-grade carriers that synergize protein hydrophobicity with pectin’s interfacial stabilization [80,81,82]. For example, quercetin, a polyphenolic compound with lipid-modulating potential, has limited application due to poor stability and low bioavailability [83]. For instance, a black kidney bean protein isolate–pectin–quercetin complex (0.1:3:1 mass ratio) achieved 94.72% encapsulation efficiency and reduced premature release during digestion, overcoming quercetin’s inherent instability and low bioavailability [84]. Similar food-grade polysaccharide/protein delivery platforms, such as pectin/alginate emulgel particles and alginate/chitosan-coated zein nanoparticles, have also been shown to protect lipophilic bioactives, improve bioavailability, and enable targeted intestinal or colonic release [85,86]. In addition, plant proteins such as amaranth protein can be used to fabricate nanoparticle-based delivery systems with good encapsulation and controlled-release properties [87]. These studies support carrier-mediated stabilization and controlled release, but they do not by themselves prove enhanced lipid-lowering efficacy.

Some MFH-derived polysaccharides and oligosaccharides may also function dually as carriers and prebiotics, selectively promoting beneficial gut bacteria that produce short-chain fatty acids (SCFAs) linked to improved lipid metabolism [88]. Alginate oligosaccharides and inulin are representative examples: alginate oligosaccharides are derived from marine MFH resources, whereas inulin from chicory root is a classic prebiotic fiber [89,90,91,92]. Both can be selectively fermented by gut bacteria, promote beneficial microbial populations, and contribute to host metabolic homeostasis [93]. Furthermore, pectin oligosaccharides and pectin-rich byproducts have shown selective stimulation of beneficial gut bacteria and prebiotic activity comparable to inulin or fructo-oligosaccharides, while inulin-type fructans have repeatedly been linked to favorable microbiota modulation and changes in inflammatory markers [94,95,96]. This prebiotic mechanism is biologically plausible and supported by microbiota studies, but its direct contribution to lipid-lowering remains incompletely established unless paired with lipid outcomes. 

Thus, colloidal delivery systems may improve the formulation performance of MFH bioactives, while certain carbohydrate-based carriers may additionally provide microbiota-mediated benefits [97]. Because most evidence remains at the formulation, digestion, or microbiota-modulation level, future studies should compare free compounds, crude extracts, and colloidal formulations under matched-dose conditions and include serum lipid profiles, hepatic lipid accumulation, bile-acid metabolism, and microbiota-derived metabolites as integrated endpoints.

#### 4.3.4. Nanoparticles

Plant proteins and green biopolymers are attractive “green” carriers for food applications and can be engineered into nanoparticles with favorable encapsulation and release profiles [98]. This establishes their utility as delivery materials, but not their independent lipid-lowering mechanism.

Nanoparticles and plant-derived exosome-like nanovesicles (PELNs) represent emerging delivery platforms for MFH bioactives [99,100]. PELNs have been isolated from ginger, *Pueraria lobata*, tea leaves or flowers, grapefruit, strawberry, broccoli, ginseng, and other edible or medicinal plants [101,102,103]. Their reported advantages include protection of plant bioactives, potential cellular delivery, and modulation of inflammation, gut microbiota, and metabolic signaling [104]. However, most available studies focus on vesicle characterization, anti-inflammatory activity, microbiota modulation, or general metabolic regulation rather than direct hypolipidemic endpoints. Therefore, PELN-related mechanisms should be described as emerging and incompletely validated rather than proven lipid-lowering mechanisms.

Among MFH-derived PELNs, ginger-derived vesicles are currently the most extensively studied [105]. Nanoparticles isolated from *Zingiber officinale* Rosc. have shown hepatoprotective, anti-inflammatory, and metabolic regulatory effects in experimental models, including improvements in hyperlipidemia, hyperglycemia, and oxidative stress [106]. Oral administration of ginger PELNs restored Foxa2-mediated signaling, prevented diet-induced insulin resistance, and prolonged lifespan in mice [107]. Ginger-derived vesicles have also been used to deliver small molecules, antibodies, and nucleic acids, including doxorubicin, curcumin, and siRNA, supporting the feasibility of PELN-based delivery platforms [108,109]. Kudzu root (Gegen), the dried root of *Pueraria lobata* (Willd.) Ohwi, plays a pivotal role in TCM and modern herbology [110]. *Pueraria lobata*-derived exosome-like nanovesicles have also emerged as a promising example of medicinal-food homologous plant vesicles [111,112]. Recent studies suggest that they can regulate inflammatory phenotypes and gut microbiota-related pathways, supporting their potential use in disease modulation and food-derived nanodelivery systems [113,114]. Key compounds from Gegen root, such as puerarin, have been shown to reduce trimethylamine by inhibiting the *Prevotella copri* metabolic pathway, thereby lowering trimethylamine N-oxide (TMAO) levels, alleviating atherosclerosis and lipid metabolism disorders, and ultimately regulating lipid metabolism [115,116]. While direct evidence linking puerarin to these PELNs’ mechanisms is limited, the nanocarrier properties of *Pueraria* lobata-derived vesicles suggest their potential to enhance the delivery of bioactive compounds, warranting further exploration into their synergistic applications in disease modulation and nanodelivery systems. Additional examples from green tea, grapefruit, strawberries, broccoli, and ginseng further support the broad applicability of PELNs [117,118]. Nevertheless, evidence supporting lipid regulation by isolated compounds cannot automatically be extrapolated to plant-derived vesicles unless the vesicles themselves are tested using lipid-specific endpoints. At present, ginger PELNs have experimental support mainly from animal models, whereas *Pueraria*-derived PELNs and other plant vesicles remain largely mechanistic or delivery-platform hypotheses for lipid regulation.

The translation of PELNs is especially challenging because their regulatory classification remains uncertain, standardized characterization methods are lacking, and data on biodistribution, immunogenicity, long-term safety, scalable isolation, and batch reproducibility are still limited [119]. Future work should define vesicle size distribution, lipid/protein/RNA cargo, potency markers, purity, stability, and batch-level certificates of analysis. Side-by-side comparisons of free compounds, conventional extracts, and nanoparticle formulations are needed to determine whether these platforms provide true lipid-lowering benefit beyond improved delivery or general metabolic modulation. Until such lipid-efficacy studies are available, nanoparticle and PELN mechanisms should be considered promising but not clinically proven.

### 4.4. Function-Oriented Formulation

In MFH plant research, function-oriented formulation aims to combine bioactive constituents within the same plant or across different MFH plants to achieve complementary lipid-regulatory effects. Examples include combinations of polysaccharides with polyphenols or saponins with flavonoids, which may act through different mechanisms such as intestinal fermentability, bile-acid regulation, hepatic lipid metabolism, antioxidant protection, and intestinal barrier maintenance [120]. However, claims of synergy should not be inferred solely from improved compatibility, solubility, release behavior, or cellular uptake. Formulation synergy should be considered unproven unless the combined formulation shows greater lipid-regulatory effects than individual components, simple mixtures, and non-formulated controls under matched-dose conditions.

Several studies have illustrated this concept. For instance, a combination of mulberry leaf polyphenols and polysaccharides enhanced short-chain fatty acid production through the gut–liver axis and inhibited adipogenesis via the HMGCR/ACC signaling pathway [121]. In *Platycodon grandiflorum*, polysaccharides with different molecular weights showed complementary effects on intestinal microbiota remodeling and signaling pathway activation [122]. Other reported examples include combinations of total astragalosides with total flavonoids and cassia anthraquinones with polysaccharides, which may coordinate lipid metabolism, antioxidant defense, excretion, and intestinal barrier function [123]. Beyond intra-plant combinations, synergistic lipid-lowering effects have also been observed among different MFH plants. For example, the combination of ginsenosides and *Perilla frutescens* seed extract was reported to regulate hepatocellular lipid metabolism by activating the PI3K-Akt pathway, suppressing lipid synthesis-related genes such as DGAT2, and enhancing glucose uptake through IRS2-dependent signaling [124]. In another study, a multi-plant compound preparation composed of *Gastrodia elata*, *Emblica officinalis*, and other botanicals modulated cholesterol metabolism via the gut–liver axis and improved intestinal barrier integrity in a Caco-2/HepG2 co-culture model [125]. Cinnamon essential oil and proanthocyanidins self-assembled into nanocolloids and inhibited digestive enzymes related to glucose and lipid metabolism [126]. These examples provide mechanistic or preclinical support for multi-target regulation, but the specific molecular mechanisms responsible for clinical lipid-lowering remain uncertain until confirmed in controlled animal studies and human trials.

Overall, function-oriented formulation provides a rational strategy for multi-target lipid regulation, but most supporting evidence remains preclinical or formulation-based. Cell-based, co-culture, and in vitro digestion models provide mechanistic support, whereas direct hypolipidemic efficacy requires validation in animal models and, ultimately, randomized human trials. Future studies should include dose–response analysis, non-formulated controls, chemical standardization, toxicological assessment, herb–drug interaction evaluation, and lipid-specific outcomes such as serum TG, TC, LDL-C, HDL-C, hepatic triglyceride accumulation, fecal lipid/bile-acid excretion, SCFA production, and lipid-metabolism-related gene/protein expression. Therefore, function-oriented formulation should be viewed as an enabling strategy rather than proof of efficacy until its lipid-lowering benefit, mechanism, and safety are confirmed by appropriately controlled metabolic studies [127].

## 5. Bioactive Components and Mechanisms of Hypolipidemic Action in MFH Plants

The dual edible–medicinal nature of MFH plants makes them promising sources for hypolipidemic agents. Reported mechanisms are class-specific: polysaccharides frequently act through gut microbiota remodeling, short-chain fatty acid production, and bile-acid modulation; flavonoids and polyphenols mainly regulate hepatic AMPK/PPAR/SREBP pathways involved in lipogenesis and fatty acid oxidation; and saponins or triterpenoids may influence cholesterol transport and bile-acid pathways, including LDLR/LXR/ABCA1 and FXR signaling [128]. 

However, most mechanistic and efficacy evidence comes from in vitro and animal studies, whereas human randomized trials remain limited and heterogeneous. Therefore, this section classifies mechanisms according to evidence level: strong preclinical evidence, moderate preclinical evidence, preliminary mechanistic evidence, or limited clinical evidence. Mechanistic findings derived only from cell or animal models are interpreted as hypothesis-generating unless supported by human lipid endpoints. Detailed study attributes are summarized in Table 2, Table 3 and Table 4.

### 5.1. Gut Microbiota-Mediated Mechanisms of Hypolipidemic Action in MFH Plants

The gut microbiota provides an important link between host health, nutrient metabolism, and disease progression [163]. Because many MFH bioactive constituents are highly polar or relatively large molecules, their absorption in the upper gastrointestinal tract is often limited, and their biological effects may depend partly on microbial biotransformation and microbially derived metabolites rather than direct systemic exposure of parent compounds [164]. Preclinical studies suggest that MFH constituents can regulate lipid metabolism through a “microbiota → SCFAs/bile acids → intestinal barrier → liver” axis, in which microbial remodeling increases short-chain fatty acids, alters bile-acid pools, improves intestinal barrier integrity, and influences hepatic lipid handling [165]. This mechanism is well supported in rodent and in vitro studies, but causal confirmation in humans remains limited.

Among MFH constituents, polysaccharides are the most representative microbiota-targeting compounds. As prebiotic substrates, they have attracted increasing attention in both the pharmaceutical and functional food fields [166]. In preclinical models, polysaccharides improve lipid dysregulation through microbiota modulation; limited human data also suggest metabolic benefit [43,167]. For example, *Poria cocos* polysaccharides and water-insoluble polysaccharides from *Poria* reduced hepatic steatosis and improved lipid/glucose parameters in HFD and ob/ob mouse models, accompanied by increased intestinal butyrate and improved mucosal integrity [134,168]. *Cistanche deserticola* polysaccharides increased *Prevotella* spp., enhanced SCFA production, and activated AMPK in rodent models, coinciding with reduced hepatic lipogenesis [136,169]. *Ganoderma lucidum* polysaccharides modulated bile-acid synthesis, reverse-cholesterol-transport genes, and inflammatory/oxidative markers while reducing dyslipidemia in HFD-fed rodents [137,170]. Together, these studies show a consistent preclinical pattern linking polysaccharide-induced microbiota remodeling with improved lipid outcomes in animals.

Oligosaccharides and certain oils show microbiota-modulating activity and improved metabolic readouts in animal models, but their evidence is less consistent than for polysaccharides and remains mainly experimental. Alginate oligosaccharides reduced serum TG and LDL-C in HFD-fed mice while increasing beneficial bacteria such as *Akkermansia muciniphila*, *Lactobacillus reuteri*, and *Lactobacillus gasseri* [138]. Perilla seed oil (PSO), which is rich in polyunsaturated fatty acids, has been reported to improve high-fat diet-induced intestinal dysbiosis in mice, including in combination with metformin [171]. PSO also improved intestinal barrier integrity, reduced inflammation, and altered microbial composition, thereby helping to prevent lipid metabolic disorders [171,172]. Similarly, Laminaria japonica oil was shown to improve high-fat diet-induced gut microbial dysbiosis, increase SCFA synthesis, and help restore metabolic balance despite its low lipid content [173]. Alkaloids and quinones can also alter gut communities and hepatic signaling in rodents, yet these findings are hypothesis-generating and require human validation before clinical claims are made. In high-fat diet-induced rats, alkaloids from mulberry leaves, such as 1-deoxynojirimycin (DNJ), improved hyperlipidemia by remodeling gut microbial structure [132,174]. Nuciferine from *Nelumbo nucifera* Gaertn. and Aurantio-obtusin, a lipid-lowering active component of Cassiae Semen, have also been reported to exert anti-hyperlipidemic effects by altering gut microbial composition, including enrichment of the mucin-degrading bacterium *Akkermansia muciniphila*, and by modulating hepatic lipid metabolism-related genes [175,176]. Dietary fibers such as inulin remain classic prebiotics with established microbiota effects and plausible metabolic benefits, but fiber trials also vary in formulation and endpoints [91].

Overall, microbiota-mediated hypolipidemic mechanisms have different levels of translational support: relatively strong preclinical evidence for polysaccharides and prebiotic fibers, moderate preclinical evidence for selected oligosaccharides and oils, and preliminary evidence for alkaloids and quinones. Human validation remains limited and requires standardized interventions, predefined lipid endpoints, and integrated mechanistic biomarkers such as SCFAs, bile-acid profiles, and microbiome/metabolomic readouts. Therefore, microbiota remodeling should be interpreted as supportive mechanistic evidence, not standalone clinical proof of lipid-lowering.

### 5.2. Regulation of Hepatic Lipid Metabolism

Key hepatic regulatory nodes implicated in MFH studies include AMPK activation, PPAR-mediated fatty acid oxidation, SREBP-mediated lipogenesis, cholesterol efflux, and bile-acid turnover. These pathways summarize the major small-molecule effects reported in preclinical work. However, most evidence comes from hepatocyte models and HFD-fed animals rather than human mechanistic trials. 

Flavonoids and related small molecules are frequently associated with AMPK/PPAR/SREBP modulation [177]. Flavonoid-rich extracts from *Hippophae rhamnoides* L. increased PPAR-γ, ABCA1, and CPT1A expression, while isorhamnetin further upregulated PPAR-γ, LXRα, and CYP7A1, suggesting coordinated effects on cholesterol efflux and bile-acid synthesis [142,178]. Mogroside V from *Siraitia grosvenorii* has been shown to upregulate PPAR-α and CPT1A while downregulating SREBP1 and SCD1, thereby favoring fatty acid oxidation over de novo lipogenesis [145,179,180]. Naringin from Cassia seeds and 6-gingerol from *Zingiber officinale* also attenuate hepatic lipogenesis, mainly through suppression of SCD-related pathways and reduction in oxidative or metabolic stress [176,181]. These findings support biological plausibility but should not be interpreted as clinical proof. For example, the high PPARγ agonist activity of compound 2a from Piper nigrum remains preliminary without in vivo and clinical validation [182,183]. Similarly, cycloether terpenoid glycosides from *Cornus officinalis*, including strychnoside and moroside, have been associated with FOXO and PGC-1α/LXR signaling, but supporting evidence remains limited [184,185,186].

Several MFH plants also influence hepatic lipid accumulation through inhibition of lipogenesis, promotion of cholesterol efflux, and enhancement of bile-acid metabolism. *Exocarpium Citri Grandis* and hawthorn extract may reduce hepatic lipid accumulation by inhibiting SCD activity, suppressing pancreatic lipase, and interfering with cholesterol micelle formation [187,188]. In hawthorn extract, rutin, chlorogenic acid, and isoquercetin have been suggested as putative bioactive constituents, although their relative contributions remain to be determined [189,190]. GLPs have been reported to activate the LXRα-ABCA1/ABCG1 axis and increase CYP7A1 and CYP27A1 expression, which is consistent with enhanced bile acid synthesis and cholesterol disposal [137]. Platycodon grandiflorum saponin, particularly platycodin D, has shown cholesterol-lowering potential by increasing LDLR expression and suppressing the LXR-IDOL pathway in experimental models [191,192]. Likewise, AMBP80-1a from *Allium macrostemon* reduced cholesterol deposition in ox-LDL-stimulated THP-1 foam cells, suggesting a possible anti-atherogenic effect [193,194,195]. Restoration of hepatic insulin sensitivity may provide an additional protective mechanism [196]. *Ziziphus jujuba* improved diet-induced dyslipidemia through IRS-1/PI3K/Akt signaling, and a neutral polysaccharide from red dates reduced oleic acid-induced triglyceride accumulation in hepatocytes [197,198]. Perilla seed oil also attenuated fat storage by inhibiting lipogenic enzymes and enhancing fatty acid catabolism [199]. 

In summary, hepatic AMPK–PPAR–SREBP regulation, suppression of de novo lipogenesis, promotion of fatty acid oxidation, and modulation of cholesterol/bile-acid homeostasis are biologically plausible mechanisms supported by moderate-to-strong preclinical evidence. Their clinical relevance remains uncertain because human pharmacokinetic/pharmacodynamic data, confirmed hepatic exposure levels, and adequately powered trials with prespecified lipid endpoints are still limited.

### 5.3. Adipose Tissue Remodeling

Adipose tissue is a dynamic endocrine and metabolic organ that contributes to systemic energy homeostasis through lipid storage, lipolysis, and the secretion of bioactive mediators, making it an important therapeutic target in lipid metabolic disorders [200]. Among MFH plants, Raphani Semen, the dried mature seeds of *Raphanus sativus* L., has attracted attention because several constituents have been reported to influence adipocyte differentiation and adipose remodeling in experimental models [201]. However, the current evidence remains largely preclinical, and the contribution of individual constituents has not been fully established. 

Sulforaphane (SFN) and sulforaphene have been reported to inhibit adipocyte differentiation through Hedgehog signaling, potentially limiting adipocyte hyperplasia and obesity-related adipose expansion [202]. SFN has also been shown in preclinical studies to promote adipocyte apoptosis by modulating the Akt/p70S6K1/Bad and ERK pathways and may facilitate adipocyte browning through Nrf2/SIRT1/PGC-1α activation, a pathway associated with mitochondrial biogenesis and thermogenic programming [203,204]. 

Collectively, Raphani semen-derived compounds may influence adipose remodeling through inhibition of adipogenesis, induction of adipocyte apoptosis, and promotion of thermogenic programming. Nevertheless, these mechanisms are supported predominantly by cell-based and rodent studies. Therefore, adipose-tissue remodeling should be interpreted as preliminary-to-moderate preclinical evidence rather than clinical proof of lipid-lowering efficacy. Human validation will require standardized metabolic phenotyping, including body composition, lipolysis rates, insulin sensitivity, lipid-specific endpoints, and safety assessment.

### 5.4. Antioxidant and Anti-Stress Effects

Oxidative stress and chronic low-grade inflammation contribute to metabolic dysfunction [205]. Many MFH plants reduce reactive oxygen species, lipid peroxidation, inflammatory activation, and stress-related signaling in experimental models [6]. However, antioxidant or anti-inflammatory activity should be regarded as a supportive mechanism rather than direct evidence of clinically meaningful lipid-lowering. Improvements in oxidative or inflammatory biomarkers are insufficient to establish hypolipidemic efficacy unless accompanied by prespecified lipid outcomes such as TG, TC, LDL-C, HDL-C, or hepatic lipid accumulation [206]. 

Several MFH-derived compounds illustrate this supportive mechanism. *Perilla frutescens* (L.) Britt. contains diverse bioactive constituents, and its ethyl acetate and ethanol extracts have been reported to suppress ROS generation and protect against lipid peroxidation, suggesting a potential role in mitigating oxidative stress-related liver injury [207,208,209]. *Portulaca oleracea* L. is rich in flavonoids that may support lipid metabolism [180]. Among its constituents, quercetin has been associated with beneficial effects in metabolic syndrome and lipid abnormalities through antioxidant, anti-inflammatory, and lipid-modulating pathways [144]. Crocin, a natural food colorant derived from saffron (*Crocus sativus* L.), has also been reported to exert anti-inflammatory and antioxidant effects [210]. A meta-analysis suggested that crocin supplementation may reduce fasting blood glucose and total cholesterol, but the available clinical evidence is still limited and should be interpreted cautiously [211]. Mechanistically, crocin has been linked to the DAF-16/FOXO pathway in experimental systems, which may help prevent lipid peroxidation-induced vascular endothelial injury [212]. Astragaloside IV (AS-IV), a major bioactive component of *Astragalus membranaceus*, also exhibits immunomodulatory and antioxidant activities, although its specific contribution to lipid-lowering still requires stronger validation [213].

Importantly, oxidative stress and inflammation often act together in the pathogenesis of cardiometabolic disorders, including obesity, type 1 and type 2 diabetes, and hyperlipidemia, as well as related conditions such as obstructive sleep apnea [214]. In this context, *Ganoderma lucidum* polysaccharides have been reported to ameliorate HFD-induced hyperlipidemia by suppressing TLR4/MyD88/NF-κB signaling in adipose tissue [215]. Overall, antioxidant and anti-inflammatory effects may complement primary lipid-regulatory mechanisms, particularly hepatic and gut-mediated pathways, but they do not independently prove sustained lipid-lowering. Future studies should combine oxidative/inflammatory biomarkers with lipid-specific endpoints to clarify clinical relevance. 

### 5.5. Dose–Response, Cytotoxicity, and Adverse Effects

The hypolipidemic effects of MFH plants are generally dose- and concentration-dependent, but the effective range varies across species, extracts, and preparation methods because phytochemical composition and extraction conditions differ substantially [216,217]. Safety reporting remains heterogeneous, and many studies rely on short-term observation rather than systematic toxicological evaluation.

Several examples illustrate the need for product-specific safety assessment. Artichoke leaf extract has been studied at 600 mg/day and 2700 mg/day in divided doses, but caution is advised during pregnancy/lactation and in patients with bile duct obstruction or gallstones; most reported adverse reactions are mild gastrointestinal complaints, although rare hepatotoxicity has been described [217]. Red yeast rice carries statin-like safety risks: because monacolin K is chemically identical to lovastatin, the product may cause liver, muscle, and kidney adverse effects, and commercial preparations show marked lot-to-lot compositional variability, with occasional citrinin contamination [218,219]. In contrast, short-term clinical trials of combined supplements containing red yeast rice and artichoke leaf extract did not identify clinically relevant abnormalities in liver, renal, or muscle safety parameters, suggesting that acceptable tolerability is possible under controlled conditions [220]. High-dose licorice extracts may cause glycyrrhizin-related toxicity, while American ginseng may interact with drugs such as warfarin and potentially affect bleeding risk [221,222].

Overall, safe and effective human dosing data remain limited for most MFH plants and cannot be inferred solely from cell or animal studies. Future evaluations should address dose–response relationships, long-term tolerability, contaminant control, herb–drug interactions, and toxicological profiles before broader clinical translation.

### 5.6. Clinical Evidence and Translational Relevance

Although in vitro and animal studies support the lipid-regulatory potential of MFH plants, human evidence remains limited, heterogeneous, and unevenly distributed across interventions. Therefore, the translational relevance of MFH-derived products should be evaluated according to evidence level, reproducibility of lipid endpoints, preparation standardization, and safety monitoring. At present, most MFH-derived polysaccharides, flavonoids, saponins, alkaloids, quinones, and plant-derived nanoparticle systems remain supported mainly by preclinical evidence, whereas only a few MFH-related interventions have been assessed in randomized trials or systematic reviews.

Red yeast rice-containing products currently have the strongest clinical support among MFH-related lipid-lowering interventions. A systematic review and meta-analysis reported that red yeast rice extract reduced serum lipid parameters in humans with hypercholesterolemia [218]. However, because monacolin K is chemically identical to lovastatin, clinical use requires strict standardization, contaminant testing, dose control, and safety monitoring [218,219]. 

Clinical evidence is also available for selected combined formulations [223]. A supplement containing standardized extracts of amla, walnut, olive, and red yeast rice reduced LDL-C and remnant cholesterol levels in patients with hypercholesterolemia [33]. In addition, a four-month randomized, double-blind, placebo-controlled trial showed that nattokinase–Monascus supplementation improved dyslipidemia-related parameters [220]. These findings suggest that multi-component formulations may have clinical lipid-lowering potential, but attribution of efficacy to individual components or mechanisms remains difficult.

Some individual MFH-related compounds have entered clinical evaluation, but the evidence remains preliminary. Crocin improved inflammatory markers and selected cardiometabolic indices in women with polycystic ovary syndrome, and a meta-analysis suggested possible reductions in fasting blood glucose and total cholesterol [135,211]. Artichoke leaf extract has also been discussed in relation to lipid management and safety, but caution is required in individuals with bile duct obstruction, gallstones, pregnancy, or lactation [217].

Overall, current clinical evidence is strongest for selected red yeast rice-containing products and certain multi-component formulations. Most individual MFH-derived compounds and plant-derived nanoparticle systems remain supported mainly by preclinical evidence. Therefore, clinical claims should be limited to interventions tested in human studies with defined preparations, adequate controls, and prespecified lipid endpoints. Cell- and animal-derived mechanisms should be regarded as hypothesis-generating unless supported by human lipid outcomes. At the current stage, MFH plants are best positioned as promising functional food or nutraceutical resources rather than validated substitutes for established lipid-lowering therapies. 

## 6. Conclusions and Perspectives

MFH plants are widely used for both edible and medicinal purposes and show promise in hyperlipidemia management. Technologies such as microencapsulation, colloidal carriers, and nanoparticle processing may improve bioavailability, stability, and formulation performance, but their contribution to lipid-lowering efficacy requires validation using lipid-specific endpoints. Standardization of MFH plant extracts remains challenging due to differences in regulations, plant sources, growing conditions, and extraction methods. These variations lead to inconsistencies in the composition and concentration of bioactive compounds, hindering quality control and reproducibility.

In terms of clarifying the mechanism of lipid-lowering, most research evidence on MFH plants is based on in vitro experiments or animal models, while human clinical trials remain relatively scarce, making it difficult to confirm the efficacy and safety of MFH preparations. Currently, there are no clear conclusions regarding the safety of long-term, high-dose administration or use in specific populations.

Future research on MFH plants should include clinical trials to verify efficacy and dosage; in-depth study of lipid-lowering mechanisms; evaluation of the interaction of compatible ingredients; and discovery of new bioactive compounds and optimization of existing formulations using omics and bioinformatics techniques.

In summary, MFH plants demonstrate significant potential as natural medicines and functional foods. However, standardization, safety assessment, and clinical validation remain key challenges to their widespread application. Future translation will require stronger standardization, safety assessment, and clinical validation before MFH plants can be widely adopted in hyperlipidemia management.

## Figures and Tables

**Table 1:** Representative MFH plant species and their regulatory status across selected jurisdictions.

Common Name	Latin Name	Parts	GSFA	FNFC	ARTG	EFSA	Primary Basis for Inclusion
Adlay	*Coix lacryma-jobi* L.	Fruit		√	√	√	HE, PR
Angelica root	*Angelica sinensis* (Oliv.) Diels	Root		√	√	√	HE, PR
Armeniacae Semen Amarum	*Prunus armeniaca* L.	Seed		√	√		SC, PR
Astragalus root	*Astragalus membranaceus* (Fisch.) Bge.	Root			√		HE
Black pepper	*Piper nigrum* L.	Fruit	√		√	√	PR
Black sesame seed	*Sesamum indicum* L.	Seed		√	√	√	HE, PR
Chicory	*Cichorium intybus* L.	Root			√	√	HE, PR
Chinese yam	*Dioscorea opposita* Thunb.	Root	√	√	√		PR
Chinese hawthorn	*Crataegus pinnatifida* Bge.	Fruit			√		HE, PR
Chrysanthemum	*Chrysanthemum morifolium* Ramat.	Flower		√	√	√	HE, PR
Cistanche	*Cistanche deserticola* Y. C. Ma	Stems			√		HE, RC
Jujube	*Ziziphus jujuba* Mill.	Fruit		√	√		HE
Kudzu root	*Pueraria lobata* (Willd.) Ohwi	Root				√	HE, PR
Lotus	*Nelumbo nucifera* Gaertn.	Leaf; seed		√	√	√	PR
Luo han guo	*Siraitia grosvenorii* (Swingle) C.	Fruit					RC, PR
Mulberry	*Morus alba* L.	Leaf; Seed		√	√	√	HE, PR
Perilla	*Perilla frutescens* (L.) Britt.	Leaf; Seed; Stems		√	√	√	HE, PR
Sea buckthorn	*Hippophae rhamnoides* L.	fruit			√		HE, PR
Sour orange	*Citrus aurantium* L.	Flower			√		RC, SC
Turmeric	*Curcuma longa* L.	Rhizome		√	√	√	HE, PR
White mustard	*Brassica juncea* (L.) Czern. et Coss.	Seed				√	HE, PR

Abbreviations: GSFA, General Standard for Food Additives; FNFC, Foods with Nutrient Function Claims; ARTG, Australian Register of Therapeutic Goods; EFSA, European Food Safety Authority; RC, regulatory coverage; SC, safety concerns; PR, processing relevance; HE, published hypolipidemic evidence.

**Table 2:** Polysaccharides and Prebiotic-Like Components of MFH Plants and Their Hypolipidemic Mechanisms.

Compound Class	Source Plant	Study Model	Dose and Durations	Main Hypolipidemic Outcome	Primary Mechanism/Pathway	Reference
Chinese yam polysaccharide (CYP)	*Dioscorea Opposita* Thunb.	In vivo	10, 20 mg/kg/day, 8 weeks	Improved obesity-related metabolic parameters	Inhibit the levels of interleukin-10 (IL-10) and leptin in serum and the expression of matrix metalloproteinase-3 (MMP-3) and NF-kappa B (NF-κB) P65 in white adipose tissue	[129]
*Crataegus pinnatifida* polysaccharides (CPPs-H-1)	*Crataegus pinnatifida* Bge.	In vivo; In vitro	200, 400 mg/kg/day, 8 weeks; 0.25–8 mg/mL, 10 min	Enhanced lipid oxidation	Improve the peroxisome proliferator-activated receptor alpha (PPARα) pathway to promote the oxidation of fatty acids	[130]
*Lycium barbarum* polysaccharide (LBP)	*Lycium barbarum* L.	In vivo	200 mg/kg/day, 4 weeks	Improved dyslipidemia-related indices	Regulate bacterial diversity	[131]
Mulberry leaf polysaccharides (MLP)	*Morus alba* L.	In vivo	100, 200, 400 mg/kg/day, 10 weeks	Improved fat and carbohydrate metabolism	Use the gut microbiota-bile acids metabolic pathway to control the metabolism of fats and carbohydrates	[132]
Polysaccharide fraction of radish greens (PRG)	*Raphanus sativus* L.	In vivo	4 mg/kg/day, 12 weeks	Increased lipid metabolism-related protein expression	Increase in the lipid metabolism-related protein expression	[133]
*Poria cocos* polysaccharides (PCP)	*Poria cocos* (Schw.) Wolf	In vivo; In vitro	50, 100, 200 mg/kg/day, 4 weeks; 200 μg/mL, 24 h	Slowed down weight gain, hyperlipidemia, and liver steatosis; reduced pathological damage in the liver and intestines.	Improved hyperlipidemia and hepatic steatosis by inhibiting PARP-1-mediated intestinal macrophage pyroptosis, preserving intestinal barrier integrity, and reducing endotoxin translocation.	[134]
Yam starch	*Dioscorea alata* L.	In vivo	4 weeks	Decreased TG, TC, and LDL-C concentrations	Modulation ameliorates lipid metabolism in association with gut microbiota modulation	[135]
*Cistanche deserticola* polysaccharides (CDP)	*Cistanche deserticola* Y. C. Ma	In vivo; In vitro	24, 48, 96 mg/kg/day, 2 weeks; 50 μg/mL, 24 h	Improved lipid metabolism and liver injury	Modulated gut microbiota, increased SCFAs and hepatic lipid metabolism	[136]
Ganoderma lucidum polysaccharides (GLPs)	Ganoderma lucidum	In vivo	100, 200, 400 mg/kg/day, 16 weeks	Ameliorated dyslipidemia	Activated Nrf2-Keap1 pathway and inhibited NF-κB signaling. Modulated AMPK/PPARα and SREBP1C pathways to suppress lipogenesis. Promoted RCT via LXRα-ABCA1/ABCG1 signaling. Promoted BA synthesis and inhibited the intestinal FXR-FGF15 pathway	[137]
Alginate oligosaccharides (AOs)	Algal-derived oligosaccharides	In vivo	10 weeks	Reduced serum TG and LDL-C levels; Inhibited expression of lipogenesis genes; Reduced fasting blood glucose; Increased serum insulin; Lowered inflammatory markers	Gut microbiota remodeling; Significantly increased concentrations of short-chain fatty acids; Decreased levels of endotoxin	[138]

**Table 3:** Small-Molecule Bioactive Constituents of MFH Plants and Their Hypolipidemic Mechanisms.

Compound	Source Plant	Study Model	Dose and Duration	Main Hypolipidemic Outcome	Primary Mechanism/Pathway	Reference
Total flavone of *Astragalus membranaceus* (TFA)	*Astragalus membranaceus* (Fisch.) Bge	In vivo	10, 20 mg/kg/day; 16 weeks	Reduced atherosclerotic lesion size and plaque vulnerability; Ameliorated hepatic steatosis; Improved serum lipid profile; Reduced foam cell formation.	Inhibit the expression of miR-33/ABCA1/G1Pathway and NF-κB pathway	[139]
EUL 50	*Eucommia ulmoides* Oliv.	In vitro	25, 50, 100 μM/24 h	Improved lipid-regulatory activity	Regulate peroxisome proliferator-activated receptor gamma (PPARγ) expression through inducing autophagy	[140]
Naringenin, naringin, neohesperidin	*Citri grandis* (L.) Osbeck	In vivo	2.5, 5, 10 g/kg/day; 12 weeks	Reduced lipid accumulation	Anti-lipogenic and anti-iron accumulation effects	[141]
Sea buckthorn flavonoids	*Hippophae rhamnoides* L.	In vitro	40 μM/24 h	Suppressed de novo cholesterol synthesis	Promoted fatty acid oxidation	[142]
Total flavonoids	*Crataegus pinnatifida* Bge.	In vivo; In vitro	-	Reduced hepatic lipid synthesis	Downregulation of lipid synthesis-related proteins	[143]
Quercetin	*Portulaca oleracea* L.	In vitro	100 μM/24 h	Inhibited lipid synthesis	Protein kinase B/mammalian target of rapamycin/sterol regulatory element binding protein 1(AKT/mTOR/SREBP1) pathway inhibition	[144]
1-Deoxynojirimycin (DNJ)	*Morus alba* L.	In vivo	50 mg/kg/day; 8 weeks	Improved hyperlipidemia	Gut microbiota modulation	[145]
Nuciferine	*Nelumbo nucifera* Gaertn.	In vivo; In vitro	12.5, 25, 50 mg/kg/day; 4 weeks; 12.5–200 μM/24 h	Ameliorated metabolic disorders and hyperlipidemia	Modulate intestinal TAS2R46 to prevent metabolic problems and regulate hepatic very low-density lipoprotein (VLDL) metabolism	[146]
Alkaloid extract of Codonopsis radix	*Codonopsis pilosula* (Franch.) Nannf.	In vivo	50, 100, 150 mg/kg/day; 12 weeks	Reduced hepatic lipid accumulation	AMPK/PGC-1α pathway activation	[147]
Ferulic acid	*Perilla frutescens* (L.) Britt.	In vivo; In vitro	25, 50, 100 mg/kg/day; 6 weeks; 0.1, 1, 10 μM, 6 h	Improved lipid metabolic disorder	Activate acyl-coenzyme A synthetase long-chain family member 1 (ACSL1)	[148]
Caffeic acid	*Perilla frutescens* (L.) Britt.	In vivo	30, 60 mg/kg/day; 4 weeks	Reduced hepatic lipid accumulation	Keap1/Nrf2 pathway activation	[149]
Chlorogenic acid	*Taraxacum mongolicum* Hand.-Mazz.	In vivo	12 weeks	Reduced intracellular cholesterol levels	NPC1L1 and HMGCR inhibition	[150]
Catechins	*Perilla frutescens* (L.) Britt.	In vivo	576 mg/kg/day; 24 weeks	Improved fatty acid metabolism	Control the liver’s fatty acid metabolism enzymes’ RNA and protein expression to influence their function	[151]
Rosmarinic acid	*Perilla frutescens* (L.) Britt.	In vivo	100 mg/kg/day; 8 weeks	Improved antioxidant capacity	Improve the antioxidant capacity of cells	[152]

**Table 4:** Lipid-Based, Terpenoid, and Other Representative Bioactive Constituents of MFH Plants and Their Hypolipidemic Mechanisms.

Compound	Source Plant	Study Model	Dose and Duration	Main Hypolipidemic Outcome	Primary Mechanism/Pathway	Reference
α-linolenic acid	*Perilla frutescens* (L.) Britt.	In vivo	500 mg/kg/day, 64 days	Improved hyperlipidemia and metabolic disorders	Improve high-fat-diet-induced metabolic disorders and gut microbiota disorders to various degrees	[153]
Linoleic acid	*Coix lacryma-jobi* L. var. mayuen	In vivo	30.84, 61.67, 308.35, 616.70 mg/kg/day; 8 weeks	Improved hyperlipidemia	Reduces fatty acid synthase (FAS) and increases AMP-activated protein kinase (AMPK) and lipoprotein lipase (LPL)	[154]
ω-3 fatty acid	*Perilla frutescens* (L.) Britt.	In vivo	59.7 g/100 g perilla oil; 16 weeks	Improved inflammatory symptoms	Anti-inflammatory effect	[155]
Astragaloside IV	*Astragalus membranaceus* (Fisch.) Bge.	In vitro	50–200 μM/24 h	Reduced lipid buildup and lipogenesis	AMPK signaling pathway activation, lipid buildup and lipogenesis inhibition, and reduced ER stress	[156]
Oleanolic acid	*Ophiopogon japonicus* (L. f.) Ker-Gawl.	In vivo	25, 50, 100 mg/kg/day	Improved metabolic dysfunction and hepatic steatosis	Restored gut-liver axis homeostasis via repairing the intestinal barrier, re-shaping gut microbiota, and suppressing TLR4-mediated inflammation	[157]
Mogroside V	*Siraitia grosvenorii* (Swingle) C.	In vivo; In vitro	25, 50, 100 mg/kg/day; 15, 30, 60,120 μM/24 h	Reduced hepatic steatosis and lipid accumulation	Activation of the AMPK signaling system reduces fat buildup and improves hepatic steatosis	[158]
Glycyrrhizic acid	*Glycyrrhiza uralensis* Fisch.	In vitro	0~10 μM/24 h	Reduced TG accumulation and adipogenesis	Inhibit the initial phase of adipogenesis by suppressing the MAPK kinase (MEK)/ERK-mediated enhancer binding protein (C/EBP) β expression	[159]
Crocin	*Crocus sativus* L.	In vivo	30 mg/day; 12 weeks	Reduced inflammatory markers	Inhibits IL-6 and TNF-α	[160]
Sesamin	*Sesamum indicum* L.	In vivo	40, 80, 160 mg/kg/day; 7 weeks	Improved lipid metabolism and kidney injury	Enhances lipid metabolism and lessens kidney damage brought on by problems with lipid metabolism	[161]
Hawthorn fruit acid	*Crataegus pinnatifida* Bge.	In vivo; In vitro	30, 100 mg/kg/day; 8 weeks; 0.125, 0.25 mg/mL, 18 h	Attenuated oxidative damage	Inhibits oleic acid (OA)-induced oxidative damage to the human hepatocellular carcinoma cell lines (HepG2) by activating the nuclear factor-E2-related factor 2/heme oxygenase-1 (Nrf2/HO-1) signaling pathway	[162]

## Data Availability

The data presented in this study are available on request from the corresponding author.

## Abbreviations

The following abbreviations are used in this manuscript:

MFH	Medicine-food homology
SCFAs	Short-Chain Fatty Acids
TMAO	Trimethylamine-N-oxide
ROS	Reactive Oxygen Species
PUFAs	Polyunsaturated Fatty Acids
HFD	High-Fat Diet
PPAR	Peroxisome Proliferator-Activated Receptor
SREBP	Sterol Regulatory Element-Binding Protein
AMPK	AMP-Activated Protein Kinase
FAS	Fatty Acid Synthase
TLR4	Toll-Like Receptor 4
HepG2	Human Hepatocellular Carcinoma Cells
NPC1L1	Niemann-Pick C1-Like 1
GSFA	General Standard for Food Additives
FNFC	Foods with Nutrient Function Claims in Japan
ARTG	Australian Register of Therapeutic Goods
EFSA	the European Food Safety Authority

## Appendix A

**Table A1:** MFH plant species and their regulatory status across selected jurisdictions.

Common Name	Latin Name	Parts	GSFA	FNFC	ARTG	EFSA
Adlay	*Coix lacryma-jobi* L.	Fruit		√	√	√
Adzuki bean	*Vigna angularis* Ohwi et Ohashi	Seed		√		
American Ginseng	*Panax quinquefolius* L.	Root			√	
Amla	*Phyllanthus emblica* L.	Fruit			√	
Angelica root	*Angelica sinensis* (Oliv.) Diels	Root		√	√	√
Armeniacae Semen Amarum	*Prunus armeniaca* L.	Seed		√	√	
Astragalus root	*Astragalus membranaceus* (Fisch.) Bge.	Root			√	
Balloon flower root	*Platycodon grandiflorus* (Jacq.) A. DC.	Root				√
Barley malt	*Hordeum vulgare* L.	Seed			√	
Black pepper	*Piper nigrum* L.	Fruit	√		√	√
Black sesame seed	*Sesamum indicum* L.	Seed		√	√	√
Cardamom	*Amomum tsaoko* Crevost et Lemaire	Fruit				√
Cassia bark	*Cinnamomum cassia* Presl	Cortex			√	
Cassia seed	*Cassia obtusifolia* L.	Fruit				
Chameleon plant	*Houttuynia cordata* Thunb.	Whole herb				√
Chicory	*Cichorium intybus* L.	Root			√	√
Chinese angelica	*Angelica dahurica* (Fisch. ex Hoffm.) Benth. et Hook. f.	Root				
Chinese date	*Ziziphus jujuba Mill.* var. *spinosa* (Bunge) Hu ex H. F. Chou	Seed				
Chinese flowering quince	*Chaenomeles speciosa* (Sweet) Nakai	Fruit			√	
Chinese foxglove root	*Rehmannia glutinosa* Libosch.	Root				
Chinese hawthorn	*Crataegus pinnatifida* Bge.	Fruit			√	
Chinese mosla	*Mosla chinensis* Maxim.	Aboveground				√
Chinese raspberry	*Rubus chingii* Hu	Fruit			√	
Chinese torreya seed	*Torreya grandis* Fort.	Seed				
Chinese white olive	*Canarium album* Raeusch.	Fruit				√
Chinese yam	*Dioscorea opposita* Thunb.	Root	√	√	√	
Chrysanthemum flower	*Chrysanthemum morifolium* Ramat.	Flower		√	√	√
Cistanche	*Cistanche deserticola* Y. C. Ma	Stems			√	
Citron	*Citrus medica* L.	Fruit			√	√
Clove	*Eugenia caryophyllata* Thunb.	Flower	√	√	√	√
Cochinchinese asparagus root	*Asparagus cochinchinensis* (Lour.) Merr.	Root				
Cogon grass rhizome	*Imperata cylindrica* Beauv. var. *major* (Nees)C. E. Hubb.	Rhizome				
Dang shen	*Codonopsis pilosula* (Franch.) Nannf.	Root			√	
Hemp seed	*Cannabis sativa* L.	Fruit			√	
Hua ju hong	*Citrus grandis* (L.) Osbeck	Exocarp				
Hyacinth bean	*Dolichos lablab* L.	Seed			√	
Japanese apricot	*Prunus mume*(Sieb.) Sieb. et Zucc.	Fruit		√		
Japanese bush cherry seed	*Prunus japonica* Thunb.	Seed				
Japanese honeysuckle flower	*Lonicera japonica* Thunb.	Flower				√
Japanese pagoda tree flower	*Sophora japonicum* L.	Flower and flower buds			√	√
Jujube	*Ziziphus jujuba* Mill.	Fruit		√	√	
Kencur	*Kaempferia galanga* L.	Root			√	√
Kombu	*Laminaria japonica* Aresch.	Seaweed		√		
Kudzu root	*Pueraria lobata* (Willd.) Ohwi	Root				√
L. gracile	*Lophatherum gracile* Brongn.	Leaves				√
Lablab Semen Album	*Dolichos lablab* L.	Seed; Flower			√	
Lesser galangal	*Alpinia officinarum* Hance.	Root				√
Licorice	*Glycyrrhiza uralensis* Fisch.	Root				
Lily bulb	*Lilium brownii* F. E. Brown var. *viridulum* Baker	Scale leaf				
Long pepper	*Piper longum* L.	Fruit			√	
Longan	*Dimocarpus longan* Lour.	Aril				
lotus	*Nelumbo nucifera* Gaertn.	Leaf; seed		√	√	√
Luo han guo	*Siraitia grosvenorii* (Swingle)C.	Fruit				
Maidong	*Ophiopogon japonicus* (L. f.) Ker-Gawl.	Tuberous Root				
Malva nut	*Sterculia lychnophora* Hance	Seed				√
Mandarin orange	*Citrus reticulata* Blanco	Fruit				√
Mongolian dandelion	*Taraxacum mongolicum* Hand. -Mazz	Whole herb				√
Mint	*Mentha haplocalyx* Briq.	Aboveground		√	√	√
Mulberry	*Morus alba* L.	Leaf; Seed		√	√	√
Nutmeg	*Myristica fragrans* Houtt.	Seed	√			
Polygonatum	*Polygonatum sibiricum* Red.	Root				√
Poria	*Poria cocos* (Schw.) Wolf	Sclerotium			√	√
Purslane	*Portulaca oleracea* L.	Aboveground				
Radish seed	*Raphanus sativus* L.	Seed			√	√
Red Sword Bean	*Canavalia gladiate* (Jacq.) DC.	Root				
Reed rhizome	*Phragmites communis* Trin.	Root				√
Sea buckthorn	*Hippophae rhamnoides* L.	fruit			√	
Sha ren	*Amomum villosum* Lour.	Seed				√
Sharp-leaf galangal	*Alpinia oxyphylla* Miq.	Seed				√
Sichuan pepper	*Zanthoxylum bungeanum* Maxim.	Peel of fruit				
Solomon’s seal	*Polygonatum odoratum* (Mill.) Druce	Root				√
sour orange	*Citrus aurantium* L.	Flower			√	
Star anise	*Illicium verum* Hook. f.	Fruit				√
Thistle	*Cirsium setosum* (Willd.) MB.	Aboveground				
Tianma	*Gastrodia elata* Bl.	Scale leaf				
Turmeric	*Curcuma longa* L.	Rhizome		√	√	√
White mustard	*Brassica juncea* (L.) Czern. et Coss.	Seed				√

Abbreviations: GSFA, General Standard for Food Additives; FNFC, ‘Foods with Nutrient Function Claims’ in Japan; ARTG, Australian Register of Therapeutic Goods; EFSA, the European Food Safety Authority; √: Indicating the presence of this botanical in the index or ingredient databases.
